# Heat stress-mediated oxidative damage in male germ cells: potential protective effects of L-citrulline

**DOI:** 10.3389/fendo.2026.1800681

**Published:** 2026-03-20

**Authors:** Yan Qin, Hongmei Wu, Linfeng Mo, Huihuang Shen, Yanqing Tan, Weijun Li, Yonghua He, Wei Peng

**Affiliations:** 1 Department of Health Management, Guangzhou Huashang Vocational College, Guangzhou, China; 2 Department of Epidemiology and Health Statistics, Guilin Medical University, Guilin, China; 3 Department of Hospital Management, Hangzhou Children’s Hospital, Hangzhou, China; 4 Medicine and Health Science College, Guangzhou Huashang College, Guangzhou, China; 5 Department of Medical Insurance and Price, Medical Center Hospital of Qionglai City, Chengdu, China

**Keywords:** antioxidants, heat stress, L-citrulline, male infertility, nitric oxide, oxidative stress, sperm motility

## Abstract

Male factors contribute to approximately half of all infertility cases globally, with heat stress recognized as a major environmental determinant of impaired male reproductive function. Extensive research indicates that heat stress disrupts spermatogenesis through multiple pathways, including testicular oxidative stress (OS), compromise of the blood−testis barrier, and dysregulation of the spermatogonial stem cell niche. As global temperatures rise, the prevalence of heat−induced reproductive impairment is increasing, underscoring the urgent need to identify safe and effective interventions that target the underlying oxidative damage. L-citrulline demonstrates significant potential in the field of male reproductive protection. However, existing reviews primarily focus on general discussions of antioxidants, lacking a systematic analysis of the specific mechanisms of L-citrulline. This review systematically synthesizes current knowledge on the molecular mechanisms of heat stress−induced oxidative injury in male gametes. Particular emphasis is placed on the multifaceted protective role of L−citrulline, which acts through synergistic mechanisms involving modulation of the arginine−nitric oxide (NO) pathway, preservation of mitochondrial homeostasis, restoration of autophagy flux, and suppression of apoptotic signaling. By integrating experimental and clinical evidence, this analysis aims to elucidate both the translational potential and the key scientific challenges of L−citrulline supplementation in male reproductive health. The review seeks to advance the translation of L−citrulline from basic research to clinical practice and to propose novel nutritional strategies for improving fertility outcomes in men exposed to heat stress.

## Graphical Abstract

**Figure:**
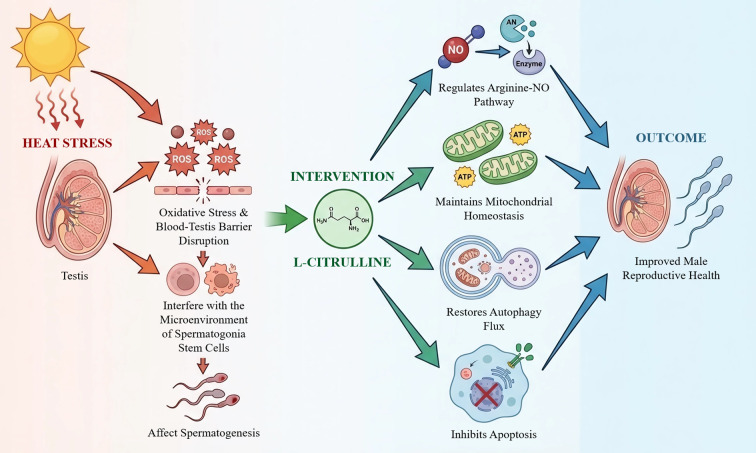

## 1 Introduction

Infertility impacts more than 10% of couples worldwide, with compromised semen quality representing a major contributing factor in male partner (1, 2). Among environmental determinants, heat stress is a well-established risk factor for diminished semen quality in men (3). Normal spermatogenesis and sperm motility are critically dependent on the maintenance of testicular temperature at 3–5 °C below core body temperature. Elevations in ambient or local scrotal temperature can disrupt this thermal gradient, inducing testicular heat stress and associated dysfunctions, including impaired sperm motility (4, 5). Epidemiological findings by Zhou et al. established a dose-response relationship between ambient temperature and semen quality (6). Specifically, a 5 °C temperature increase was associated with reductions in sperm concentration (0.70 × 10^6^/mL), total sperm count (4.09 × 10^6^), and both total and progressive motility (1.01% and 1.06%, respectively). Importantly, heat-induced impairment of spermatogenesis exhibits a progressive worsening with prolonged exposure, underscoring the cumulative threat posed by sustained thermal stress to male reproductive function (5). Preclinical animal studies conducted in models such as boars, rams, and mice corroborate these findings. They demonstrate that heat stress impairs reproductive performance through diminished semen quality and testicular histological damage (5, 7–10). Moreover, such thermal stress may also compromise the efficacy of assisted reproductive technologies (ART) (11).

Of the diverse pathological alterations elicited by heat stress, oxidative stress (OS) is widely regarded as a central mechanism responsible for the deterioration of sperm quality (6, 12, 13). OS arises when the dynamic equilibrium between intracellular reactive oxygen species (ROS) production and clearance is disrupted, leading to excessive ROS accumulation that overwhelms endogenous antioxidant defenses. This redox imbalance subsequently inflicts oxidative damage on essential biological macromolecules, including lipids, proteins, and DNA (14), with the male reproductive system exhibiting particular vulnerability to such harm (15, 16). Heat stress-induced OS impairs testicular steroidogenesis by disrupting key enzymatic pathways, thereby suppressing testosterone synthesis and compromising the function of both Sertoli and Leydig cells. Furthermore, it disrupts critical phases of spermatogenesis, including spermatocyte meiosis and spermiogenesis. Collectively, these insults manifest as significant declines in semen parameters, notably sperm concentration, motility, and morphology (17–21). Concurrent systemic adaptations to heat stress—such as peripheral vasodilation and hypoglycemia resulting from elevated glucose uptake—further increase cellular stress (22). These systemic changes act synergistically with localized OS to exacerbate reproductive dysfunction. Therefore, elucidating the molecular mechanisms underlying ROS overgeneration and OS imbalance is not only a critical prerequisite for clarifying heat stress-induced male reproductive damage, but also provides a theoretical foundation for subsequent investigations into the protective effects of L-citrulline through targeted intervention in the aforementioned pathways.

L-citrulline is a non-essential amino acid synthesized primarily within intestinal enterocytes from glutamine via ornithine transcarbamylase catalysis (23). Following its release into the systemic circulation, L-citrulline is transported to the kidneys, where it is converted into L-arginine through the sequential actions of argininosuccinate synthase and argininosuccinate lyase (24). *In vivo*, L-citrulline is chiefly metabolized through the arginine-nitric oxide (NO) pathway, yielding NO and polyamines as terminal metabolites. These bioactive molecules are capable of modulating key signaling cascades, including the mitogen-activated protein kinase (MAPK) pathway (25). Given the established role of NO in systemic thermoregulation (26, 27), the contribution of its precursor, L-citrulline, to NO biosynthesis and thermal homeostasis has emerged as a significant research focus (28, 29). Notably, the protective effects of L-citrulline against heat stress-related reproductive damage are achieved precisely by targeting the aforementioned key pathological processes, including OS and mitochondrial dysfunction. Existing studies indicate that L-citrulline, via an NO-dependent mechanism, can inhibit the mitochondrial translocation of dynamin-related protein 1 (Drp1), thereby preventing mitochondrial fragmentation and preserving mitochondrial membrane potential under thermal challenge (28). Simultaneously, it attenuates heat stress-induced overproduction of ROS, suppresses caspase-3/7 activation, and reduces apoptotic signaling (28). Animal model studies further demonstrate that even acute L-citrulline administration can enhance testicular hemodynamics, increase testicular volume, and elevate local NO bioavailability (30). Moreover, compared to other related amino acids such as L-arginine, L-citrulline exhibits superior pharmacokinetic properties, including enhanced bioavailability and more stable plasma concentration profiles (31). These characteristics underscore its considerable translational potential as a nutritional intervention for male reproductive protection against heat stress.

While the detrimental impact of heat stress on male semen quality and the beneficial role of L-citrulline in supporting reproductive health are increasingly recognized, robust evidence remains largely confined to preclinical animal and cellular models. A systematic explanation for human studies, particularly regarding the translational pathway from molecular mechanisms to clinical interventions, is still lacking. Furthermore, existing review articles predominantly focus on general discussions of antioxidants, lacking a systematic analysis of the specific mechanisms of L-citrulline. Through a comprehensive review of the existing literature, this paper aims to synthesize current knowledge on the effects of heat stress on sperm quality and to critically analyze the pathophysiological relationships linking heat stress, L-citrulline metabolism, and semen parameters. Particular emphasis will be placed on exploring the potential mechanisms and clinical applicability of L-citrulline in preserving human sperm function, with the goal of advancing its translation into evidence-based strategies for male reproductive health.

## 2 Pathological mechanisms of heat stress-induced sperm damage

### 2.1 Spermatogenic microenvironment disruption and resultant failure

Spermatogenesis in mammals constitutes a highly intricate and precisely orchestrated process of cellular differentiation, the fidelity of which depends upon the stability of the testicular spermatogenic microenvironment. This microenvironment is sustained by a dynamic cellular network involving Leydig cells, peritubular myoid cells (PMCs), and Sertoli cells (SCs), which collectively interact with spermatogonia to establish and maintain an optimal niche for spermatogonial stem cells (SSCs). The precise regulation of this niche is essential for ensuring normal reproductive function (32).

**Figure 1:**
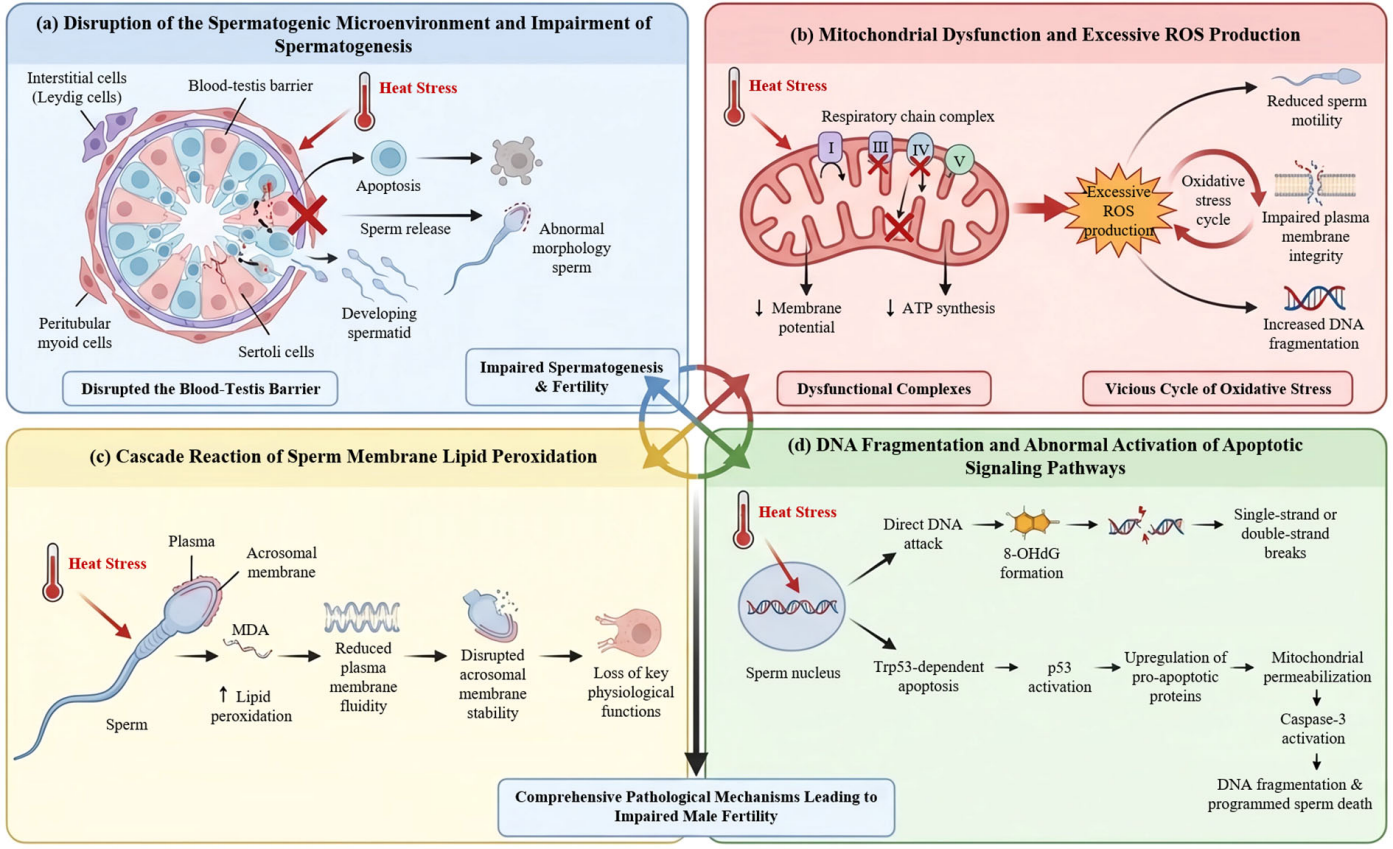
Pathological mechanism of sperm damage caused by heat stress. **(a)** Disruption of the spermatogenic microenvironment and spermatogenic dysfunction: Heat stress triggers cell apoptosis and damages the blood-testis barrier, leading to sperm morphological abnormalities. **(b)** Mitochondrial dysfunction and excessive ROS production: Heat stress inhibits the activity of mitochondrial respiratory chain complexes, resulting in decreased membrane potential, impaired ATP synthesis, and excessive ROS generation. This forms a vicious cycle of OS, ultimately compromising plasma membrane integrity and exacerbating sperm dysfunction. **(c)** Cascade of sperm membrane lipid peroxidation: Heat stress increases the level of sperm lipid peroxidation, which weakens plasma membrane fluidity, thereby destabilizing the membrane structure of the acrosomal region. This ultimately leads to the loss of key sperm physiological functions. **(d)** DNA fragmentation and abnormal activation of apoptotic signaling pathways: Heat stress-induced oxidative damage in sperm compromises DNA integrity through two pathways: 1) directly attacking DNA molecules, inducing single- or double-strand DNA breaks; 2) activating the Trp53-dependent intrinsic apoptotic pathway, initiating programmed cell death, resulting in DNA fragmentation and sperm apoptosis.

Heat stress represents a significant disruptor of testicular homeostasis, impairing spermatogenesis through multiple synergistic pathways. Research demonstrates that heat stress-induced damage is characterized by a rapid onset and progressive severity. Experimental data indicate that even brief exposure (e.g., 7 days) can result in a marked increase in sperm morphological abnormalities (33). Prolonged exposure (e.g., 14 days) exacerbates these defects, leading to compromised integrity of the sperm plasma and acrosomal membranes, accompanied by significant declines in sperm density and viability (9, 33, 34). This pathological cascade is initiated by early cellular events, including the induction of germ cell apoptosis and disruption of the blood-testis barrier (15). The consequent chronic dysregulation of the spermatogenic microenvironment ultimately culminates in aberrant spermatogenesis and diminished fertility (**Figure 1a**). Furthermore, L-citrulline blocks this pathological process by suppressing apoptosis and preserving the blood-testis barrier (35). Notably, susceptibility to heat stress exhibits considerable interspecific and intraspecific variability, including marked differences among individuals (36). This variation suggests that the extent of damage is modulated by intrinsic factors, with genetic background and intrinsic physiological homeostasis serving as key determinants. In particular, the functional efficacy of hormonal regulatory circuits and the capacity of intracellular antioxidant defense systems are critical in defining individual tolerance thresholds and pathological outcomes.

### 2.2 Mitochondrial dysfunction and excessive ROS production

Mammalian sperm motility is critically dependent on adenosine triphosphate (ATP), which fuels flagellar movement via the action of dynein ATPase (37). Mitochondria constitute the principal site of cellular energy metabolism, where oxidative phosphorylation within the inner membrane electron transport chain drives ATP synthesis (38). Beyond their bioenergetic role, mitochondria are also a physiological source of ROS, which serve essential signaling functions in processes such as cholesterol efflux, sperm capacitation, tyrosine phosphorylation, and sperm-egg interaction. However, these core functions also render mitochondria highly vulnerable to thermal insult. A growing body of evidence indicates that heat stress profoundly impairs mitochondrial physiology. It leads to a significant reduction in mitochondrial membrane potential and suppresses the activity of key respiratory chain complexes (e.g., I, III, IV, and V), thereby disrupting ATP synthesis (5, 39). This bioenergetic dysfunction is further exacerbated by the induction of excessive mitochondrial ROS production (5, 40). Consequently, a self-perpetuating cycle of OS is established, which persistently undermines sperm function. Key functional deficits resulting from this cycle include reduced progressive motility, loss of plasma membrane integrity, and increased DNA fragmentation (**Figure 1b**) (5, 9, 10, 33, 40, 41).

Therefore, interrupting this detrimental cycle necessitates targeted suppression of pathological ROS generation at its mitochondrial source. As a co-substrate for nitric oxide synthase, L-citrulline may regulate the activity of mitochondrial respiratory chain complexes by promoting endogenous NO production, thereby reducing electron leakage and excessive ROS generation (5, 28, 39). Therefore, therapeutic interventions aimed at modulating mitochondrial ROS output during heat exposure represent a promising strategy for alleviating subsequent oxidative damage to sperm (5, 40). In light of these functional insights, research emphasis is increasingly shifting from establishing phenotypic correlations to elucidating the underlying structural determinants of mitochondrial dysfunction. Studies have demonstrated a strong association between ultrastructural mitochondrial defects and diminished sperm motility (42), highlighting mitochondrial integrity as a core mechanism through which heat stress compromises sperm movement. Consequently, preserving mitochondrial architecture and stability is foundational for maintaining normative sperm motility.

### 2.3 Sperm membrane lipid peroxidation cascade

Among the diverse factors compromising sperm function, OS induced by heat stress constitutes a pivotal pathological mechanism. This central role is fundamentally attributable to the intrinsic structural vulnerability of the sperm plasma membrane. Characterized by a high concentration of polyunsaturated fatty acids (PUFAs), this membrane exhibits a high degree of fluidity—a property indispensable for sustaining sperm motility, facilitating capacitation and the acrosome reaction, and thereby establishing a critical foundation for successful fertilization (43). Paradoxically, the unsaturated bonds that confer membrane fluidity also represent primary targets for oxidative attack, predisposing the membrane to peroxidation and consequent functional loss (44). Empirical data confirm that lipid peroxidation (LPO) levels in sperm exhibit a significant increase between 7 and 14 days following heat stress exposure (33), concurrent with the accumulation of various peroxidative by-products (33, 40). The ramifications of LPO extend well beyond mere chemical alteration. It initiates a deleterious cascade, commencing with a reduction in membrane fluidity and stability, progressing to the disruption of acrosomal integrity, and culminating in the impairment of core sperm physiological functions (**Figure 1c**) (45). Of particular significance is the observation that LPO levels demonstrate a delayed peak, becoming most pronounced at 21 days post-heat stress (33). This temporal pattern underscores that oxidative damage represents not merely an acute stress response, but rather a progressive and cumulative pathological process (5). L-citrulline may preserve sperm membrane integrity by mitigating cumulative membrane lipid peroxidation through direct scavenging of free radicals or enhanced regeneration of endogenous antioxidants such as glutathione (30, 46).

While the mechanisms of heat stress-induced oxidative injury are under active investigation, parallel research focus has expanded to encompass the endogenous protective and regulatory systems within sperm. Notably, during human sperm capacitation, a marked upregulation of heat shock protein 90 (Hsp90) occurs. This molecular chaperone is critically involved in regulating several key processes: the maintenance of intracellular calcium homeostasis, the facilitation of tyrosine phosphorylation, and the mediation of progesterone signaling pathways (47). These multifunctional roles position Hsp90 as a promising molecular target for therapeutic strategies aimed at ameliorating sperm dysfunction under stress conditions.

### 2.4 DNA fragmentation and aberrant activation of apoptotic signaling

The integrity of sperm DNA is a fundamental prerequisite for successful fertilization and healthy embryonic development. Structural alterations in sperm DNA can directly compromise gene expression and protein function, thereby reducing fertilization potential (48). OS induced by heat exposure is a primary mechanism underlying the disruption of this DNA integrity. Empirical studies have confirmed that heat stress promotes oxidative damage, leading to increased sperm DNA fragmentation and elevated apoptotic markers. Detailed investigations indicate that heat stress induces a delayed but significant rise in the sperm DNA fragmentation index (49, 50) and further activates molecular apoptotic pathways, notably through enhanced caspase-3 activity (51). These effects are fundamentally linked to the excessive generation of ROS under thermal stress (12, 52). Consequently, such damage impairs the fertilizing capacity of sperm and poses potential risks to normal embryogenesis (53). The mechanistic basis of heat stress–induced sperm DNA damage involves two principal pathways. The first is direct oxidative assault, characterized by the formation of 8-hydroxy-2’-deoxyguanosine (8-OHdG) (54) and DNA strand breaks, which, if unrepaired, may lead to heritable mutations. The second pathway entails Trp53-mediated apoptotic activation (55), initiating programmed cell death. This process begins with DNA damage–induced p53 activation, which upregulates pro-apoptotic proteins, induces mitochondrial outer membrane permeabilization, and culminates in caspase-3–mediated execution, resulting in characteristic DNA fragmentation and sperm death (**Figure 1d**). L-citrulline may preserve sperm DNA integrity by attenuating direct oxidative DNA damage via reduced mitochondrial ROS and suppressing Trp53-dependent apoptotic pathway activation through maintained mitochondrial membrane potential.

Beyond direct genotoxic effects, heat stress has also been shown to significantly alter sperm epigenetic profiles, including miRNA expression patterns (55). These modifications suggest that paternal epigenetic inheritance may influence embryonic development, with potential long-term implications for offspring health. Additionally, notable inter−breed differences exist in susceptibility to heat stress–induced DNA damage (10). This variation not only informs practical breeding strategies but, more fundamentally, underscores genetic background as a key determinant of OS intensity. Such insights provide a critical theoretical foundation for the long-term genetic enhancement of thermotolerance in animal populations.

## 3 Metabolic characteristics and functional basis of L-citrulline

### 3.1 The metabolic pivotal role of the arginine-NO pathway

**Figure 2:**
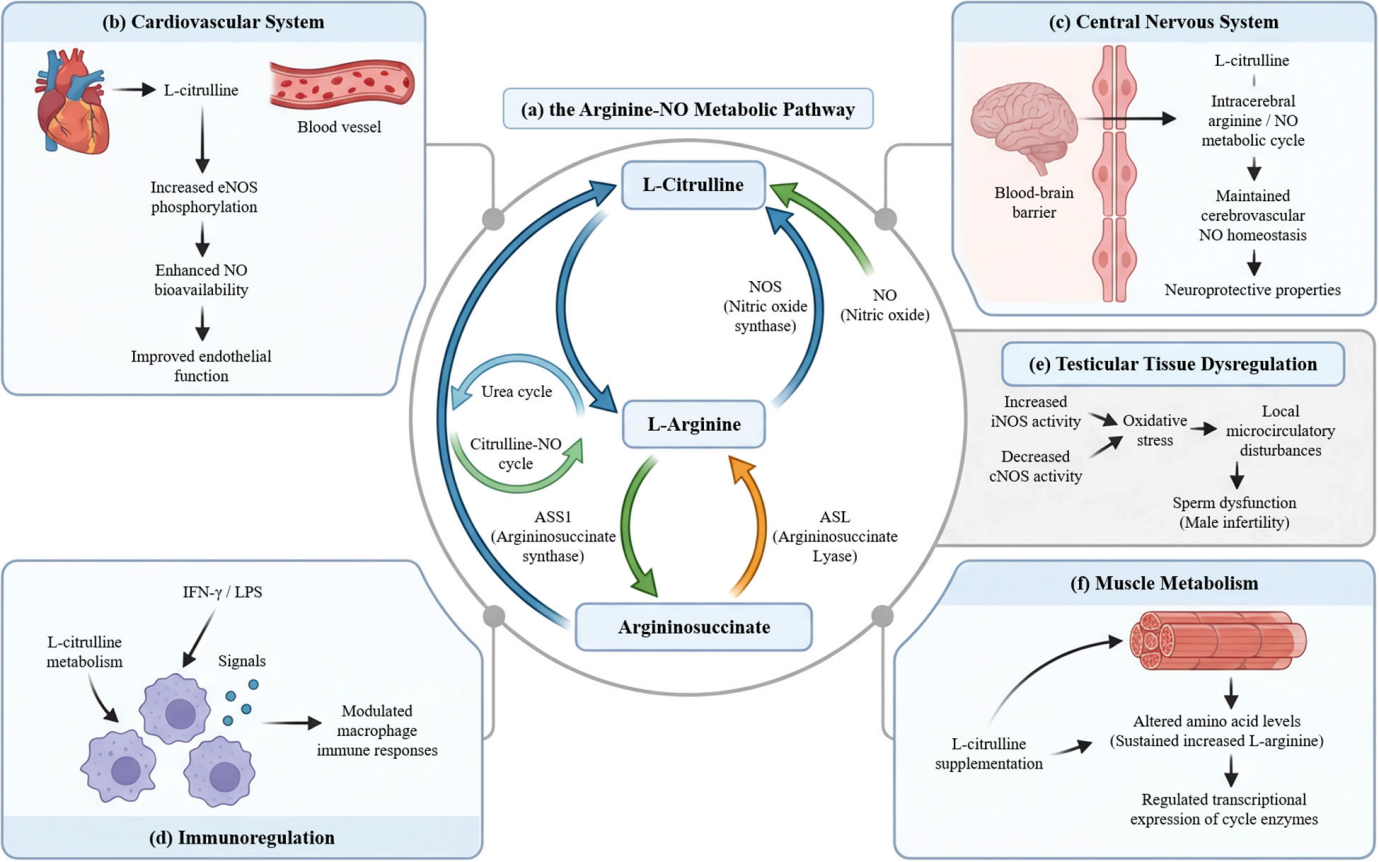
Metabolic characteristics of L-citrulline and its functional basis. **(a)** The hub function of the arginine-NO pathway: The nitric oxide synthase (NOS)-catalyzed conversion of L-arginine to L-citrulline and NO constitutes a complete metabolic cycle. **(b)** Cardiovascular system: L-citrulline improves endothelial function by enhancing the phosphorylation activity of endothelial nitric oxide synthase (eNOS), thereby increasing NO bioavailability. **(c)** Central nervous system: L-citrulline crosses the blood-brain barrier, participates in the intracerebral arginine/NO metabolic cycle, and exerts neuroprotective effects. **(d)** Immune regulation: L-citrulline can modulate macrophage immune responses under IFN-γ/LPS stimulation. **(e)** Testicular tissue: Heat stress induces increased iNOS activity, leading to oxidative stress and local microcirculatory dysfunction; L-citrulline exerts protective effects by providing substrate for NO. **(f)** Muscle metabolism: L-citrulline supplementation alters amino acid levels in muscle tissue, maintains L-arginine concentration, and regulates the transcriptional expression of urea cycle enzymes, supporting muscle energy metabolism.

L-citrulline serves as a pivotal intermediate in the arginine–NO metabolic pathway, functioning as a direct precursor for L-arginine. This cyclic pathway, catalyzed by NO synthase (NOS), facilitates the continuous conversion of L-arginine to L-citrulline and NO (**Figure 2a**) (25, 56, 57). Pharmacokinetically, L-citrulline exhibits distinct advantages by circumventing extensive hepatic first-pass metabolism, thereby promoting a more stable and prolonged elevation of systemic L-arginine concentrations. Consequently, L-citrulline supplementation demonstrates superior bioavailability compared to direct L-arginine administration in clinical settings (58–60). Metabolically, L-citrulline is converted to L-arginine via a two-step enzymatic process involving argininosuccinate synthase (ASS1) and argininosuccinate lyase (ASL). This critical transformation operates at the intersection of the urea cycle and the citrulline–NO cycle (56, 61). Owing to this unique metabolic route, L-citrulline represents an optimal therapeutic target for modulating NO signaling, with considerable potential in conditions characterized by endothelial dysfunction (25, 62, 63). Notably, this metabolic axis holds significant pathophysiological relevance within the male reproductive system. Dysregulation of the citrulline–NO cycle in testicular tissue is closely linked to impaired sperm function. A characteristic manifestation involves an upregulation of inducible NOS (iNOS) activity concomitant with a decline in constitutive NOS (cNOS) activity. This imbalance exacerbates OS and disrupts local microcirculation, collectively contributing to the pathophysiology of male infertility (**Figure 2e**) (64).

### 3.2 Biological basis of multi-organ protective effects

The multi-organ protective effects of L-citrulline are principally attributed to its capacity for systemic modulation of NO signaling. Within the cardiovascular system, L-citrulline enhances NO bioavailability by promoting the phosphorylation and activation of endothelial NO synthase (eNOS), thereby improving endothelial function (**Figure 2b**) (65). This action is not isolated. L-citrulline and L-arginine demonstrate synergistic activity in promoting endothelial cell proliferation, migration, and angiogenesis (66). In clinical contexts such as coronary artery disease, this synergy results in significant improvements in flow-mediated vasodilation (67). Beyond the vasculature, L-citrulline exerts protective effects in the central nervous system. Its high permeability across the blood-brain barrier establishes it as an efficient precursor for the cerebral arginine/NO cycle. By supporting cerebrovascular NO homeostasis, citrulline demonstrates neuroprotective potential in models of cerebrovascular dysfunction and associated neurological disorders (**Figure 2c**) (68).

L-citrulline’s regulatory influence further extends to immune and metabolic systems. Immunologically, L-citrulline metabolism participates in macrophage response modulation (69), with its intracellular concentrations being dynamically regulated by immune stimuli such as interferon-γ (IFN-γ) and lipopolysaccharide (LPS) (**Figure 2d**) (61). In muscle metabolism, L-citrulline supplementation induces a coordinated metabolic shift: it significantly alters the tissue concentration profile of eight amino acids, consistently elevates L-arginine levels, and synchronously modulates the transcriptional expression of key enzymes within the urea and NO cycles (**Figure 2f**) (70). Collectively, through these multi-organ and multi-level regulatory interactions, L-citrulline establishes a robust pharmacological basis for its therapeutic application in OS-related pathologies (62).

## 4 Antioxidant protective mechanisms of L-citrulline

### 4.1 Activation of the arginine-NO pathway

**Figure 3:**
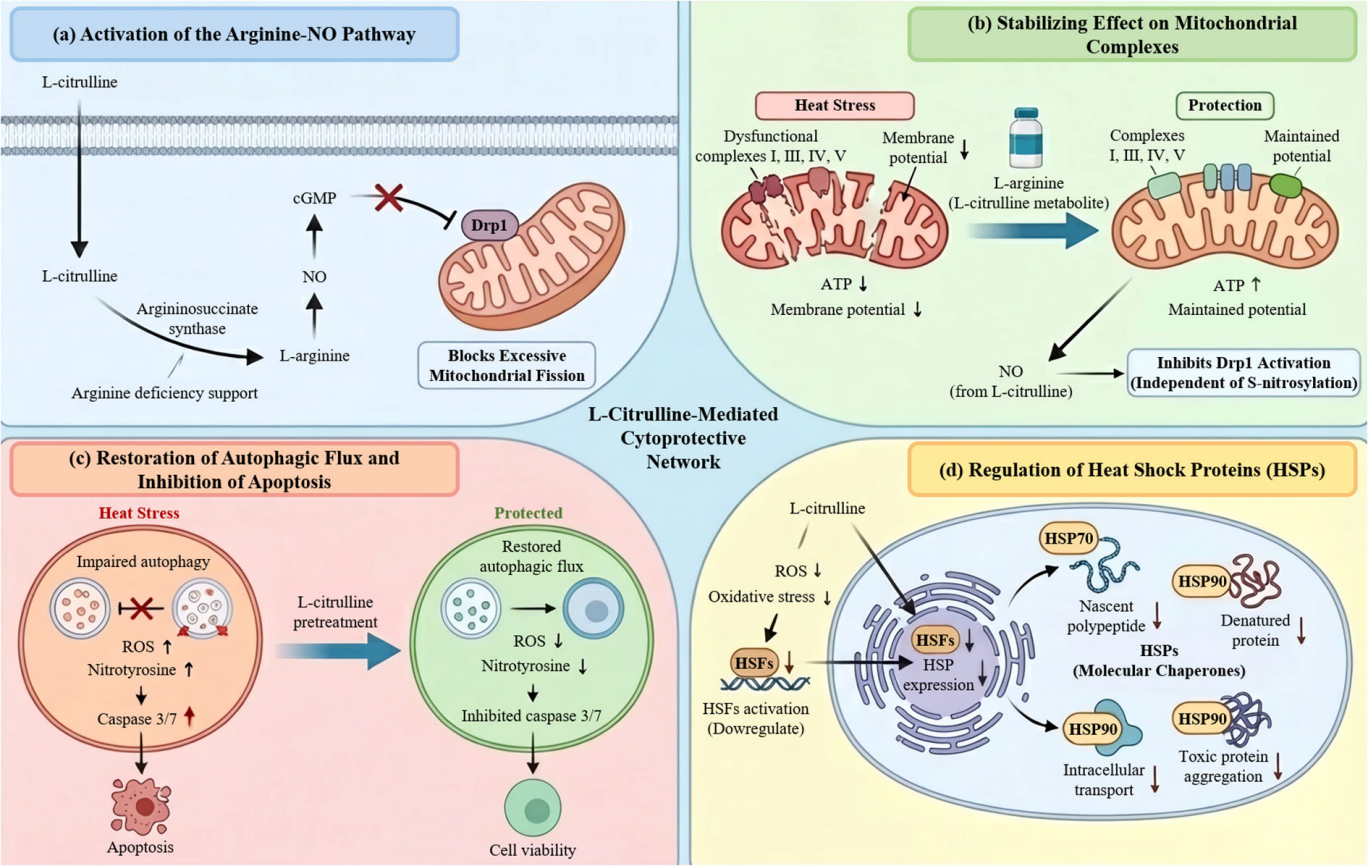
Antioxidant protective mechanism of L-citrulline. **(a)** NO-dependent inhibition of Drp1 translocation. **(b)** Stabilizing effect on mitochondrial respiratory chain complexes. **(c)** Restoration of autophagic flux and suppression of apoptosis. **(d)** Inhibition of heat shock factors to regulate heat shock proteins expression.

L-citrulline mediates its principal protective effects predominantly through the arginine-NO pathway. Pretreatment with L-citrulline has been shown to significantly elevate intracellular NO concentrations (28). This upregulation activates the downstream cGMP signaling cascade, which in turn suppresses the mitochondrial translocation of dynamin-related protein 1 (Drp1) (**Figure 3a**), thereby effectively attenuating pathological mitochondrial fission. Critically, the protective effects of L-citrulline are entirely abolished by co-administration of the NO synthase (NOS) inhibitor L-NAME (28). While NO signaling is likely a key mediator in this process, the potential involvement of L-citrulline in other pathways, such as polyamine synthesis, cannot be excluded. It is noteworthy that the metabolic utility of L-citrulline is especially evident under pathophysiological conditions. During states of arginine deficiency, L-citrulline functions as an efficient substrate to sustain NO synthesis via its conversion to L-arginine through the argininosuccinate synthase pathway (71).

Translational clinical research provides robust evidence for the efficacy of L-citrulline supplementation. In a randomized controlled trial of patients with type 2 diabetes, L-citrulline administration significantly increased serum levels of nitrite/nitrate (NOx), a stable marker of NO production (72). Furthermore, studies in heart failure patients demonstrate that long-term L-citrulline supplementation improves vascular endothelial function and enhances exercise capacity (73). Mechanistic analyses attribute these benefits directly to improve systemic NO bioavailability. Similarly, in sickle cell disease, L-citrulline therapy has shown clinical utility by ameliorating vascular function and reducing the incidence of vaso-occlusive crises (74). Collectively, these findings consistently indicate that L-citrulline exerts therapeutic benefits across diverse disease models through the enhancement of NO signaling. Its capacity to improve endothelial function, increase tissue perfusion, and alleviate clinical symptoms underscores the clinical potential of L-citrulline as a modulator of the NO pathway in various cardiovascular and related disorders.

### 4.2 Stabilizing effects on mitochondrial complexes

L-citrulline confers significant protection to mitochondrial respiratory chain complexes. Under heat stress (39 °C), spermatozoa exhibit mitochondrial dysfunction characterized by diminished membrane potential, impaired ATP synthesis, and a marked reduction in the activities of respiratory chain complexes I, III, IV, and V (5, 39). Supplementation with L-arginine, a direct metabolic derivative of L-citrulline, effectively counteracts these deficits and helps sustain physiological levels of energy metabolism in sperm (**Figure 3b**) (5). Although this finding is based on L-arginine administration, it supports the rationale for L-citrulline intervention, given the well-established precursor-product relationship between L-arginine and L-citrulline. Parallel investigations in C2C12 myoblasts further demonstrate that L-citrulline treatment provides dual protection against heat stress (43 °C). It not only preserves mitochondrial network integrity and normal ultrastructure but also functionally prevents excessive mitochondrial fragmentation (28). Mechanistically, this cytoprotective action is mediated through an NO-dependent inhibition of Drp1 activation, via a pathway independent of protein S-nitrosylation (**Figure 3b**) (28). This delineates a novel regulatory mechanism for L-citrulline in mitochondrial dynamics, wherein it indirectly modulates Drp1 activity through upstream NO signaling rather than via direct post-translational modification. Functionally, the resulting suppression of pathological fission stabilizes the structural architecture of mitochondrial complexes, thereby ensuring sustained electron transport chain efficiency, as evidenced in sperm models (5, 39, 75). The translational relevance of this mechanism is underscored by findings from a diabetic animal model, wherein L-citrulline supplementation significantly improved skeletal muscle mitochondrial function, enhanced the activity of complex IV, and increased overall oxidative phosphorylation capacity (76). Collectively, these data indicate that exogenous L-citrulline supplementation may constitute a viable therapeutic strategy to augment cellular tolerance to thermal stress, bearing considerable physiological and pathophysiological significance. These outcomes not only advance our mechanistic understanding of L-citrulline’s role in cellular bioenergetics but also furnish critical experimental rationale and novel directions for developing therapeutic interventions targeting mitochondrial dysfunction.

### 4.3 Restoration of autophagic flux and apoptosis inhibition

Autophagy is a highly conserved, lysosome-dependent catabolic process essential for the maintenance of cellular homeostasis. It involves the formation of double-membraned autophagosomes that sequester cytoplasmic cargo—including macromolecules, damaged organelles, or pathogens—for delivery to lysosomes and subsequent degradation. This pathway plays a critical role in recycling cellular components and promoting cell survival under various stress conditions (77–79). Autophagy induction can be triggered by diverse cellular signals, such as nutrient deprivation, OS, infection, or the accumulation of misfolded proteins. Emerging research indicates that L-citrulline exerts cytoprotective effects through the coordinated regulation of autophagic flux and apoptotic signaling. Under conditions of 43 °C heat stress, pretreatment with L-citrulline significantly suppresses caspase-3/7 activation, reduces apoptosis, and enhances cell viability. More critically, it restores impaired autophagic flux, effects that are closely linked to its ability to preserve NO metabolic homeostasis (**Figure 3c**) (28, 80). The underlying protective mechanisms involve the inhibition of excessive ROS production (5, 28), attenuation of mitochondrial membrane potential loss (5, 39), and reduction in the formation of nitrative stress markers, such as nitrotyrosine (71). By mitigating oxidative and nitrative damage at its source, these fundamental antioxidant effects have been corroborated in an intestinal ischemia-reperfusion model, where citrulline supplementation was shown to alleviate tissue oxidative damage and inflammatory responses (63). Furthermore, L-citrulline demonstrates protective effects at a more precise level of cellular regulation. Mechanistically, it restores NO bioavailability, thereby counteracting asymmetric dimethylarginine (ADMA)-mediated nitrative stress and preserving normal autophagic flux in airway epithelial cells (28, 71). This regulatory mechanism facilitates a more profound level of cytoprotection (**Figure 3c**).

### 4.4 Regulation of heat shock proteins expression

The heat shock response constitutes an evolutionarily conserved cellular defense mechanism against diverse stressors, including thermal, chemical, and oxidative insults (81). Central to this protection is the induced expression of heat shock proteins (HSPs), which are ubiquitously present across prokaryotes and eukaryotes (82). Functioning as molecular chaperones, HSPs participate in essential processes such as nascent polypeptide folding, refolding of denatured proteins, facilitation of intracellular transport, and prevention of toxic protein aggregation (**Figure 3d**) (83, 84). Depending on the nature, duration, and intensity of the stress, cells activate specific protective pathways, primarily through the general stress response and the hyperthermic stress response (85). The hyperthermic stress response, in particular, is highly conserved and, beyond its role in stress protection, modulates various normal cellular functions (86). As central effectors of this system, HSPs establish an integrated cytoprotective network through multiple mechanisms: the maintenance of proteostasis, regulation of macromolecular complex assembly and disassembly (87), inhibition of heat-induced protein misfolding, facilitation of protein repair and nascent peptide folding (88), and activation of anti-infective immunity (**Figure 3d**) (89–91).

Emerging evidence indicates that specific metabolites can play pivotal roles in modulating HSP expression. For instance, under conditions of environmental heat stress, supplementation with L-citrulline was shown to significantly improve hemodynamic parameters in camel testicular tissue, as evidenced by increased testicular blood flow, tissue volume, and elevated NO levels (30). These improvements are closely associated with the antioxidant properties of L-citrulline, which not only scavenges excess ROS but also potentiates intracellular antioxidant defense systems, thereby mitigating heat stress-induced oxidative damage (**Figure 3d**). It is noteworthy that this antioxidant effect may be, at least in part, associated with the heat shock response pathway (29). Preliminary studies showed that L-citrulline intervention reduced the levels of cellular stress response markers, such as HSP-70, in mouse skeletal muscle (92). It has been hypothesized that this phenomenon may stem from L-citrulline reducing the cellular dependence on chaperone proteins by alleviating the extent of OS. Nevertheless, direct experimental evidence linking L-citrulline to the regulation of heat shock protein expression remains limited, and the underlying mechanisms warrant further investigation. As a key intermediate in arginine metabolism, whether and how L-citrulline participates in the regulation of HSPs expression warrants further in-depth investigation.

## 5 Current research controversies and clinical translation challenges

Current research on the protective effects of L-citrulline against heat stress-induced spermatogenic damage relies predominantly on animal models or *in vitro* cell systems (**Table 1**) (7, 30, 40, 46). These investigations consistently indicate that L-citrulline supplementation ameliorates semen quality parameters by modulating the NO pathway and bolstering cellular antioxidant defenses. However, significant translational gaps exist, hindering the application of these findings to clinical practice. The primary limitations can be categorized as follows.

**Table 1:** Therapeutic effects of arginine/citrulline on heat stress-induced male reproductive damage.

Author	Research subject	Study model	Intervention dose/method	Main findings	Research innovation	Major limitations
Cai et al.(2024) (93)	Porcine Sertoli Cells	*In Vitro* Cell Model	Exogenous Glutamate (700 μM, a L-citrulline cycle-related amino acid) added prior to heat stress (44 °C for 30 min)	Glutamate effectively suppressed heat stress-induced Sertoli cell apoptosis by activating the Trx1-Akt pathway and enhancing endogenous antioxidant defenses	Shifted the protective target from spermatogenic cells to Sertoli cells and innovatively proposed a novel cell protection mechanism based on metabolic cooperation.	1. This study was conducted exclusively at the *in vitro* cell line level, specifically using primary Sertoli cells, which represents a significant limitation in terms of physiological relevance and generalizability. 2. The heat stress model employed a single, fixed condition (44 °C for 30 minutes), limiting the ability to assess dose- or time-dependent responses.
Li et al.(2019) (5)	Boar Sperm	*In Vitro* Cell Model	1.0 mM L-arginine for 1 hour	Effectively alleviated heat stress-induced OS and mitochondrial dysfunction by maintaining oxidative phosphorylation and ATP synthesis, thereby preserving sperm motility.	Directly targeted mitochondria, clearly revealing L-arginine’s protective role in energy metabolism at the cellular level, moving beyond the traditional antioxidant perspective.	1. Limitations of the *in vitro* study. 2. Although the article proposes that L-arginine may exert its effects via the antioxidant pathway mediated by its metabolite NO, the current study did not directly measure NO concentrations or perform reverse validation using inhibitors of NO synthase (NOS), which limits the strength of the mechanistic conclusions.
Chen et al.(2018) (8)	Boars	Animal Model	Dietary supplementation of 0.6%, 0.8%, or 1.0% L-arginine for 6 weeks	L-arginine supplementation dose-dependently increased NO production, effectively reduced scrotal surface temperature, and improved sperm motility, normal morphology rate, total sperm count, and overall seminal plasma antioxidant capacity.	Linked reproductive system improvements with body surface cooling effects, revealing that L-arginine may aid heat dissipation via NO-mediated vasodilation.	1. Lack of assessment of reproductive performance in offspring. 2. Failure to directly demonstrate a causal link in the mechanism through interventional experiments (such as the use of NO synthase inhibitors).
Zhao et al.(2022) (46)	Rams	Animal Model	Dietary supplementation of 4, 8, or 12 g L-citrulline for 70 days	L-citrulline supplementation improved sperm concentration, motility, kinematics, and mitochondrial function by increasing serum and seminal plasma levels of reproductive hormones (e.g., GnRH, Testosterone) and antioxidant markers (e.g., SOD, GSH-Px), while reducing estradiol and radical levels.	Elucidated a multi-target mechanism involving synergistic regulation of the endocrine and redox systems.	1. Lack of assessment of reproductive performance in offspring. 2. Absence of direct interventional experiments to confirm the causal relationship of the “L-citrulline → L-arginine → NO → HPG axis → spermatogenesis” cascade.
Abdelnaby et al.(2025) (30)	Male Camels	Animal Model	Single intravenous injection of L-citrulline (155 μmol/kg BW)	Improved testicular hemodynamic parameters and increased testosterone and serum NO levels under heat stress.	Acute effect study of single IV administration demonstrating rapid onset, providing new insights for clinical acute management of heat stress.	1. Lack of ultimate assessment of semen quality and fertility. 2. Failure to elucidate the specific mechanism underlying the decrease in TAC.
Wang et al.(2025) (7)	Mice	Animal Model	L-arginine (3.75 mg/mL) in drinking water for 19 days	L-arginine improved sperm quality, testicular health, and testosterone levels, while reducing inflammation.	Clearly revealed the key role of L-arginine’s anti-inflammatory effect in testicular protection, expanding the research perspective from OS to inflammation.	1. The specific mechanism of microbial flora action remains unclear. 2. There is a lack of assessment of ultimate indicators of sperm function.

### 5.1 Model limitations

Physiological differences between species represent one of the primary factors limiting the extrapolation of findings. For instance, changes in semen parameters following heat stress occur at markedly different rates in boars compared to humans (5, 8, 40). Moreover, existing animal studies predominantly utilize acute heat stress models (55), which differ from the actual scenario of humans being chronically exposed to sub-high-temperature environments. Second, intervention strategies and experimental designs in foundational studies lack clinical alignment. Preclinical research largely utilizes preventive L-citrulline administration prior to stress induction (46), whereas clinical need often centers on therapeutic interventions for existing damage. Furthermore, humans are often exposed to complex environments (e.g., heat, toxins, psychological stress), while the protective effects of L-citrulline under combined stress conditions have not been systematically evaluated. Limitations such as differences in spermatogenic cycles and variations in heat stress exposure patterns make it difficult to directly replicate animal model findings in humans. This highlights the necessity for future research to establish more standardized preclinical protocols and to conduct rigorously designed clinical studies.

### 5.2 Dose standardization

In the translational process from animals to humans, pharmacokinetics and efficacy assessment represent two major challenges. Preclinical studies employ highly variable citrulline dosages, including dietary incorporation (0.6%-1%) (8, 29), bolus supplements (4–12 g) (46), or *in vitro* concentrations (1–2 mM) (5, 28), with no established dose-response relationship. In clinical translation, route of administration requires special consideration. While animals typically receive dietary supplementation (8, 29) or single doses (30), human males may require formulations and regimens better suited for long-term use.

### 5.3 Efficacy evaluation

Regarding outcomes, while current research effectively documents improvements in intermediate endpoints like sperm motility, mitochondrial membrane potential, and DNA fragmentation (5), it lacks long-term follow-up on critical functional outcomes such as fertilization competence and embryonic developmental potential. Drawing parallels from clinical trials of antioxidants like Menevit, which assessed live birth rates (12), future L-citrulline studies must incorporate similarly rigorous reproductive endpoint analyses.

Current research on L-citrulline’s protective mechanisms remains largely confined to conventional biomarkers, such as OS markers (e.g., ROS, SOD) and mitochondrial function parameters (5, 28, 93). This narrow focus has limited a comprehensive understanding of the integrated molecular regulatory networks underlying its effects. Future advances in the field will require a deeper mechanistic exploration and the adoption of more holistic research strategies. Given that emerging evidence indicates heat stress alters sperm miRNA expression (55), it is plausible that epigenetic regulatory mechanisms play a key role in the protective effects of L-citrulline. However, the current body of evidence regarding L-citrulline is predominantly derived from animal models, with human trial data being notably absent—a critical impediment to clinical translation. This translational gap is attributable to several formidable barriers: foremost, ethical considerations are paramount, as fertility-related drug interventions demand thorough evaluation of potential risks to offspring. Additionally, the substantial resources required for trials employing live birth rate as a primary endpoint—characterized by high costs and extended durations—pose significant logistical challenges. Furthermore, the inherent difficulty in recruiting suitable cohorts, particularly individuals with reproductive injury attributable to heat stress, further exacerbates the complexity of clinical investigation. Consequently, rigorously designed, multicenter human trials represent an indispensable prerequisite for establishing the clinical efficacy of L-citrulline and advancing its translational potential.

### 5.4 Gut-testis axis

In recent years, the gut microbiota-testicle axis has recently emerged as a critical frontier in reproductive biology. Animal studies indicate that L-arginine—a metabolite intimately linked to the citrulline-NO cycle—can alleviate testicular injury through modulation of the gut microbial ecosystem (7). This finding raises the possibility that L-citrulline may also confer indirect benefits via microbiota-mediated mechanisms, potentially influencing systemic inflammatory tone, metabolic homeostasis, and testicular function. Building on this foundation, it is hypothesized that L-citrulline exerts its reproductive protective effects through modulation of the gut microbiota via two distinct yet potentially complementary pathways. Firstly, L-citrulline and its derivatives, notably nitric oxide, may reinforce intestinal epithelial integrity, thereby attenuating the translocation of pro-inflammatory agents such as lipopolysaccharide into the systemic circulation. This reduction in low-grade systemic inflammation would, in turn, optimize the testicular microenvironment for spermatogenesis. Secondly, L-citrulline may function as a preferential metabolic substrate for commensal bacteria, fostering the expansion of beneficial populations, particularly those capable of generating short-chain fatty acids. These microbiota-derived metabolites, upon entering the circulatory system, may directly or indirectly influence testicular function and homeostasis. These hypotheses present promising avenues for elucidating the multifaceted mechanisms underlying L-citrulline’s action.

To address current research limitations and drive the field forward, the systematic application of multi-omics technologies is essential. Integrated metabolomic, proteomic, and single-cell transcriptomic profiling of testicular tissue and spermatozoa will provide a systems-level perspective on the complex biological responses to heat stress and L-citrulline intervention. In parallel, focused investigation into the gut microbiome—particularly the functional restructuring of microbial communities and associated metabolic shifts induced by L-citrulline—is necessary to elucidate the role of the microbiota-host metabolic axis in regulating reproductive health. Combining multi-omics analysis of the reproductive system with in-depth gut microbiome studies will not only uncover the systemic protective mechanisms of L-citrulline within the gut-testicle axis but also provide a scientific foundation for developing personalized, microbiome-informed nutritional strategies tailored to individual molecular phenotypes.

## 6 Conclusions

This review systematically delineates the molecular mechanisms by which heat stress impairs sperm quality through oxidative damage pathways and elucidates the multi-targeted protective role of L-citrulline in this context. Heat stress triggers a cascade of adverse effects in sperm, including mitochondrial dysfunction, loss of membrane potential, and DNA fragmentation, predominantly mediated by excessive ROS production. The core protective effects of citrulline are largely exerted through its metabolite NO, which operates via three interrelated mechanisms: first, by inhibiting the mitochondrial translocation of the fission protein Drp1, thereby preserving mitochondrial structural integrity; second, by enhancing the activity of mitochondrial respiratory chain complexes, leading to increased ATP synthesis; and third, by reducing caspase-3/7 activity and suppressing the apoptotic signaling cascade. In practical applications within animal husbandry, dietary L-citrulline supplementation has been shown to improve semen quality and reduce scrotal temperature, underscoring nutritional intervention as a viable strategy to mitigate heat stress. Collectively, these findings provide a robust scientific rationale for the translational potential of L-citrulline in male reproductive health, with modulation of the arginine–NO pathway emerging as a promising therapeutic target for enhancing sperm quality.

Nevertheless, the clinical translation of L-citrulline faces significant challenges, which necessitate focused efforts to address three key issues in future research: first, current dosage recommendations are derived predominantly from animal studies, leaving the optimal supplementation dose for human subjects yet to be determined. Second, individual variability in susceptibility to heat stress-induced damage calls for the development of personalized intervention strategies based on biomarkers of OS, such as 8-hydroxy-2’-deoxyguanosine (8-OHdG) and ROS levels. Specifically, baseline detection of 8-OHdG (a marker of DNA oxidative damage) and ROS levels in seminal plasma or serum enables patient stratification for precise intervention: For individuals with high oxidative stress (significantly elevated 8-OHdG/ROS), L-citrulline supplementation may offer greater benefit, with dose titration and efficacy monitoring guided by dynamic changes in these biomarkers. In contrast, for those with normal oxidative baseline, the necessity of intervention warrants careful evaluation. This strategy promotes a paradigm shift from uniform dosing to biomarker-guided personalized therapy, providing a feasible pathway for optimizing the precise application of L-citrulline in male reproductive health. Third, a lack of long-term safety data, particularly concerning the potential effects on sperm epigenetics, remains to be clarified. In light of these considerations, future investigations should prioritize the following directions. First, rigorously designed randomized controlled trials in human populations are needed to systematically evaluate the protective role of L-citrulline in male spermatogenesis and to explore its potential synergistic applications in assisted reproductive technologies. Second, the integration of multi-omics approaches with functional reproductive endpoints will be essential to elucidate the underlying molecular mechanisms and to decipher individual response heterogeneity. Finally, establishing interdisciplinary collaborative frameworks that bridge nutritional science, reproductive medicine, and environmental health is crucial for advancing evidence-based clinical guidelines. Only through such systematic and in-depth inquiry can clinically feasible and individually tailored therapeutic strategies be developed, thereby offering novel scientific avenues for the preservation of male fertility.

## Author’s note

To ensure accurate cultural identification, the authors' names are presented in Pinyin followed by their original Chinese characters: Yan Qin (覃艳), Hongmei Wu (吴红梅), Linfeng Mo (莫林烽), Huihuang Shen (沈慧煌), Yanqing Tan (谭演清), Weijun Li (李威君), Yonghua He (何永华), and Wei Peng (彭伟).
